# The VEGF and PEDF levels in the follicular fluid of patients co- treated with LETROZOLE and gonadotropins during the stimulation cycle

**DOI:** 10.1186/s12958-018-0367-5

**Published:** 2018-05-29

**Authors:** Jigal Haas, Rawad Bassil, Noa Gonen, Jim Meriano, Andrea Jurisicova, Robert F. Casper

**Affiliations:** 1 Division of Reproductive Sciences, University of Toronto, Lunenfeld-Tanenbaum Research Institute, Mount Sinai Hospital, Toronto, Canada; 2 TRIO fertility partners, 655 Bay St 11th floor, Toronto, ON M5G 2K4 Canada; 3 Department of Obstetrics and Gynecology, Sheba Medical Center, Sackler School of Medicine, Tel-Aviv University, Tel-Hashomer, Israel

**Keywords:** Letrozole, VEGF. PEDF

## Abstract

**Background:**

Previous studies have shown that androgens, in addition to serving as precursors for ovarian estrogen synthesis, also have a fundamental role in primate ovarian follicular development by augmentation of FSH receptor expression on granulosa cells. Recent studies have shown that aromatase inhibitor, letrozole, improves ovarian response to FSH in normal and poor responder patients, possibly by increasing intraovarian androgen levels. Studies in mice also showed an effect of letrozole to increase pigment epithelium-derived factor (PEDF) and to lower vascular epithelial growth factor (VEGF), which might be expected to reduce the risk of ovarian hyperstimulation syndrome (OHSS) with stimulation. The aim of this study was to compare the VEGF and PEDF levels in the follicular fluids of normal responders treated with letrozole and gonadotropins during the ovarian stimulation with patients treated with gonadotropins only.

**Methods:**

A single center, prospective clinical trial. We collected follicular fluid from 26 patients, on a GnRH antagonist protocol, dual triggered with hCG and GnRH agonist. The patients in one group were co-treated with letrozole and gonadotropins during the ovarian stimulation and the patients in the other group were treated with gonadotropins only. VEGF, PEDF, estrogen, progesterone and testosterone levels were measured by ELISA kits.

**Results:**

The age of the patients, the total dose of gonadotropins and the number of oocytes were comparable between the two groups. In the follicular fluid, the estrogen levels (2209 nmol/l vs. 3280 nmol/l, *p* = 0.02) were significantly decreased, and the testosterone levels (246.5 nmol/l vs. 40.7 nmol/l, *p* < 0.001) were significantly increased in the letrozole group compared to the gonadotropin only group. The progesterone levels (21.4 μmol/l vs. 17.5 *p* = NS) were comparable between the two groups.

The VEGF levels (2992 pg/ml vs. 1812 pg/ml p = 0.02) were significantly increased and the PEDF levels (9.7 ng/ml vs 17.3 ng/ml p < 0.001) were significantly decreased in the letrozole group.

**Conclusions:**

Opposite to observations in the mouse, we found that VEGF levels were increased and PEDF levels were decreased in the follicular fluid in patients treated with letrozole during the stimulation cycles. Further investigation is required to determine if patients treated with letrozole during the IVF stimulation protocol are at increased risk for developing OHSS as a result of these findings.

## Background

The addition of the aromatase inhibitor, letrozole, to gonadotropin stimulation has been an accepted treatment for oocyte retrieval in women with breast cancer and has been demonstrated to result in lower serum estrogen levels [1, 2] an endocrine response that is considered to be favourable in these women who may have estrogen sensitive tumors. In normal women undergoing IVF, lowering serum and follicular estrogen levels could also be potentially beneficial by lowering the risk of ovarian hyperstimulation syndrome (OHSS) since a correlation has been observed between administration of letrozole and lowering the incidence of OHSS [3].

A previous study in a rat model of OHSS [4] demonstrated that treatment with a single dose of letrozole on the hCG trigger day, reduced ovarian diameter, reduced VEGF levels and increased the levels of pigment epithelium derived factor (PEDF). VEGF has been identified as one of the prime causative factors in OHSS while PEDF has been shown to decrease the angiogenic activity of VEGF [5]. These combined results might be expected to reduce the risk of OHSS.

In addition, supraphysiologic levels of estrogen are believed to have a negative effect on oocyte quality [6] and embryo implantation [7] with subsequent adverse pregnancy outcomes [8, 9] and therefore, lowering estrogen levels by adding letrozole could be beneficial in normal women undergoing IVF by increasing implantation and reducing pregnancy complications.

More recently, it has also been observed that breast cancer patients undergoing IVF with the addition of letrozole have an increase in the number of oocytes recovered compared to controls [1, 2]. Several studies have shown that letrozole, improves ovarian response to FSH in normal and poor responder patients, possibly by increasing intraovarian androgen levels (1–3, 11–13). Previous studies have shown that androgens, in addition to serving as precursors for ovarian estrogen synthesis, also have a fundamental role in primate ovarian follicular development by augmentation of FSH receptor expression on granulosa cells [10].

The objective of this study was to measure VEGF and PEDF levels as well as estrogen and testosterone levels in the follicular fluids of normal responder women treated with letrozole and gonadotropins throughout the entire ovarian stimulation for IVF and to compare the results with the follicular fluid levels in patients treated with gonadotropins only.

## Methods

### Ethical approval

The study was approved by the Mount Sinai Hospital ethics review committee, and all couples were required to sign a written informed consent after the provision of complete information.

This study included a total of 26 IVF cycles performed in 26 normal responders treated at our institution between June, 2016 and March, 2017. Patients with PCOS or POR by Bologna criteria were excluded from the study.

The study group included patients on a GnRH antagonist protocol treated with daily letrozole 5 mg together with gonadotropins, from the first day of ovarian stimulation until the trigger day. The control group included patients matched by age, infertility diagnosis and starting gonadotropin dose, on the same GnRH antagonist protocol, treated with gonadotropins only during the stimulation. All the patients included in the study had a dual trigger with hCG and GnRH agonist for final oocyte maturation. The study was not randomized and the decision whether to co-treat with letrozole was made by each treating physician independently.

The decision to administer letrozole was not made for the sake of this study. The treating physicians made this treatment decision independently of the study.

### Stimulation protocols

Gonadotropin treatment (with or without letrozole) was initiated on the 3rd day of menses with the use of recombinant FSH (Gonal F, EMD Serono). Once the leading follicle had reached a size of 13 mm, or E2 levels exceeded 1200 pmol/L, co-treatment with a GnRH antagonist 0.25 mg/day (Orgalutran, Merck) and recombinant LH (Luveris, Serono) or highly purified human menopausal gonadotropin (Menopur, Ferring) was commenced. Follicle growth and hormone levels were serially monitored by ultrasound and blood tests until the dominant follicles reached an average diameter of 18–20 mm. At that point human chorionic gonadotropin (10,000 IU Pregnyl; Merck, Kirkland, Quebec) and GnRH agonist (0.5 mg Suprefact; Sanofi-Aventis, Canada) were administered subcutaneously to trigger ovulation. Thirty-six hours later oocyte retrieval was performed under transvaginal guided ultrasound and needle aspiration.

The follicular fluid (FF) was obtained at the time of oocyte retrieval for the IVF procedure. Only the first follicle from each ovary was collected avoiding blood clots, and only FF from mature full sized follicles (≥16 mm) was included in the study. Only one single follicular fluid sample per patient was included in the study.

After isolation of the oocyte, the clear follicular fluid was centrifuged at 500 g for 10 min at room temperature to separate out cellular contents and debris and was then transferred to sterile tubes and frozen at − 80 °C until analysis. The follicular fluid was analyzed for testosterone, estradiol, progesterone by an automated assay (Vitros), and VEGF (Cedarlane; CL76149K) and PEDF (Chemicon;CYT 420) concentrations were measured by commercial ELISA using an ELISA kits according to the manufacturer’s instructions. We measured follicular fluid progesterone concentrations as a possible way to ensure similar ovarian stimulation in each group.

Comparisons were performed using the paired two-tailed student’s t-test. A *P* < 0.05 was considered statistically significant.

## Results

The age of the patients, the total dose of gonadotropins and the number of oocytes were comparable between the two groups (Table 1). In the follicular fluid, the mean estrogen level (2209 nmol/l vs. 3280 nmol/l, *p* = 0.02) was significantly decreased, and the mean testosterone level (246.5 nmol/l vs. 40.7 nmol/l, *p* < 0.001) was significantly increased in the letrozole co-treated group compared to the gonadotropin only group. The mean follicular fluid progesterone level (21.4 μmol/l vs. 17.5 μmol, *p* = NS) was comparable between the two groups (Table 2).

**Table 1:** Characteristics of the IVF cycles for patients co-treated with letrozole compared to the control group

	Letrozole group (*n* = 13)	Without letrozole (n = 13)	*P* value
Age (years)	36.3 ± 3.9	35.8 ± 3.7	NS
AMH (pmol/l)	14.26 ± 7.7	16.4 ± 6.7	NS
FSH	7.3 ± 1.6	6.6 ± 1.9	NS
Etiology for infertility	Unexplained-8 Male factor-3 Mechanical-0 Fertility preservation-2	Unexplained-7 Male factor-3 Mechanical-1 Fertility preservation- 2	NS
Length of stimulation (days)	9.4 ± 1.8	10.7 ± 1.7	NS
Dosage of gonadotropins	3085 ± 633	3294 ± 917	NS
Oocytes (n)	11.7 ± 5.7	12.1 ± 6.1	NS
2PN(n)	6.6 ± 5.1	7.6 ± 4.4	NS
Blastocysts (n)	3.1 ± 2.2	2.9 ± 1.9	NS
Blastocyst rate (blast/2PN)	46.9%	38.1%	NS
E2 levels (pmol/l)	1032 ± 375	8069 ± 3068	0.001
Ongoing Pregnancy rate	5/11 (45.4%)	4/11(36.3%)	NS

**Table 2:** The hormone levels in the follicular fluid from patients co-treated with letrozole and gonadotropins vs. gonadotropins only

	Letrozole group (13)	Control group (13)	P
Estrogen(nmol/l)	2009 ± 1034	3280 ± 1371	0.01
Testosterone(nmol/l)	246.5 ± 153.2	40.7 ± 14.3	< 0.001
Progesterone(μmol/l)	21.4 ± 8.3	17.5 ± 10.3	0.3

The mean VEGF level (2992 pg/ml vs. 1812 pg/ml *p* = 0.02) was significantly increased and the mean PEDF level (9.7 ng/ml vs 17.3 ng/ml p < 0.001) was significantly decreased in the letrozole group (Table 3).

**Table 3:** The VEGF and PEDF levels in the follicular fluid from patients co-treated with letrozole and gonadotropins vs. gonadotropins only

	Letrozole group (13)	Control group (13)	p
VEGF (pg/ml)	2992 ± 431.7	1812 ± 462.4	0.02
PEDF (ng/ml)	9.7 ± 5.7	17.3 ± 8.4	< 0.001

None of the patients in the study group or in the control group developed early or late OHSS.

## Discussion

In contrast to observations in mice, we found that VEGF levels were increased and PEDF levels were decreased in the follicular fluids of patients treated with letrozole during the stimulation cycles, despite a significant suppression of estradiol concentration in follicular fluid. In the murine model, letrozole was administered only at the trigger day and not during the ovarian stimulation whereas in our current study, the patients were treated during the entire ovarian stimulation, which might explain the differences between the VEGF and PEDF levels observed.

Similarly to the murine findings, He et al. demonstrated a decrease in the VEGF serum levels after treatment with letrozole in the luteal phase. He found a dose dependent decrease in the levels of VEGF with increasing doses of letrozole administered in the luteal phase [11]. The findings of He et al. suggested that letrozole could decrease the risk of OHSS although it is not clear if the effect on VEGF and PEDF secretion was a direct action of letrozole or an indirect effect through a reduction in estradiol levels.

A randomized controlled study in hyper-responder patients which aimed to compare the efficacy of letrozole to aspirin during the luteal phase in primary prevention of early ovarian hyperstimulation syndrome showed a lower incidence of OHSS in women receiving letrozole compared with aspirin [3]. In contrast to previous studies, the patients treated with letrozole had higher levels of VEGF in the serum compared to the patients not treated with letrozole. The authors hypothesized that the mechanism of lower incidence of OHSS was independent of VEGF but rather due to the induction of a luteolytic effect and lower estradiol concentrations which reduced the risk of early-onset OHSS (5).

Although we didn’t measure the VEGF or PEDF levels in the serum, we found increased VEGF and PEDF levels in the follicular fluid of letrozole treated patients at the time of oocyte retrieval. In the follicular phase, letrozole reduces serum estrogen levels which results in reduced negative feedback on gonadotrophin secretion from the hypothalamus-pituitary axis [12–14]. By lowering serum estrogen concentrations in the early follicular phase, letrozole causes secretion of more FSH and LH, which acts directly on the granulosa cells and may be responsible for the increased secretion of VEGF. In addition, we found higher intrafollicular levels of testosterone in the letrozole group. We believe that the androgen increase may have a positive effect on follicular development, oocyte maturation and implantation. Since androgens have been shown to increase FSH receptor expression in both murine [15] and primate models [16, 17] it is possible that the increased VEGF level could also be influenced by the impact of increased androgen levels on the granulosa cell responsiveness to FSH in the letrozole treated group.

Previous studies demonstrated [18, 19] that women who did not conceive had higher FF VEGF concentrations than women achieving a clinical pregnancy. A negative correlation was observed between FF VEGF concentrations, peak estradiol levels and number of oocytes retrieved. Friedman et al. [20] showed increased VEGF levels in the FF from patients with advanced age compared with younger women. They hypothesized that the higher VEGF concentrations resulted from relative follicular hypoxia which is a stimulant for VEGF production.

As published previously by our group [21], we found a higher number of oocytes, zygotes and blastocysts in women co-treated with letrozole compared to matched patients treated with gonadotropins only. We hypothesize that in normal responders co treated with letrozole the pathophysiology increasing VEGF levels is different and not related to follicular hypoxia. We believe that by lowering serum estrogen concentrations in the early follicular phase, letrozole causes secretion of more FSH and LH, which acts directly on the granulosa cells and may be responsible for the increased secretion of VEGF.

VEGF binds to specific receptors located in endothelial cells called VEGF-R1 (Flt-1) and VEGF-R2. Soluble VEGF-R1 is a naturally produced receptor capable of binding and sequestering VEGF and is able to reduce the level of free, active VEGF [22].

Jakimiuk et al. [23] demonstrated that VEGF/ sFlt-1 ratio in FF on the day of oocyte retrieval in women undergoing IVF procedure, regardless of the type of stimulation protocol, might predict the risk of developing OHSS.

The sFlt-1 contribute to the amount of free, biological active VEGF in FF and later in serum by binding VEGF and thereby depleting the amount of free circulating biological active VEGF.

In our study we didn’t measure sFlt-1 levels in the FF and therefore it’s still speculative whether free circulating VEGF levels were different between the groups.

Tropea et al. [24], cultured human luteal phase with androgens and demonstrated that different doses of androgens significantly increased VEGF secretion. By culturing the cells with aromatase inhibitor, VEGF levels decreased. We think it’s difficult to compare those results with our current study because in our study the patients were treated with letrozole which causes secretion of LH and FSH from the pituitary gland in contrast to the cultured granulosa cells which are not affected by those changes. Another major difference is that the granulosa cells were cultured in the luteal phase whereas in our study the patients were treated with letrozole in the follicular phase and the granulosa cells in the follicular phase may respond differently than in the luteal phase to androgens in terms of VEGF production.

In the letrozole group the estrogen levels in the follicular fluid were significantly lower and more similar to the estrogen levels in the natural cycle compared to estrogen levels in the gonadotropins only group [25]. Previous studies have demonstrated a reduced pregnancy rate with increasing E2/oocyte ratio [6, 26] and therefore we assume that treatment with letrozole which reduces the serum and intrafollicular estrogen concentrations, may have a positive effect on the oocyte quality and embryo development.

Although none of the patients in our study group developed OHSS, we think that further investigation is required to determine if patients treated with letrozole during the IVF stimulation protocol are at increased risk for developing OHSS as a result of our new findings demonstrating increased VEGF levels and decreased PEDF levels after treatment with letrozole.

We conclude that co-treatment with gonadotropins and letrozole during the follicular phase increase the VEGF levels and decrease the PEDF levels in the follicular fluid. Whether co-treatment with letrozole during the follicular phase may increase the incidence of OHSS is still unknown and further studies should be performed to evaluate this risk.

## Abbreviations

ET: Embryo transfer
FET: Frozen embryo transfer
FSH: Follicle stimulating hormone
IVF: In vitro fertilization
OHSS: Ovarian hyperstimulation
PEDF: Pigment epithelium-derived factor
VEGF: Vascular epithelial growth factor
